# Molecular and functional ecology of aquatic microbial symbionts

**DOI:** 10.3389/fmicb.2013.00059

**Published:** 2013-03-15

**Authors:** Hans-Peter Grossart, Lasse Riemann, Kam W. Tang

**Affiliations:** ^1^Department of Experimental Limnology, IGB-Leibniz-Institute of Freshwater Ecology and Inland FisheriesBerlin, Germany; ^2^Institute of Biochemistry and Biology, Potsdam UniversityPotsdam, Germany; ^3^Department of Biology, University of CopenhagenCopenhagen, Denmark; ^4^Department of Biological Sciences, Virginia Institute of Marine Science, Gloucester PointVA, USA

Traditional aquatic microbial ecology has largely neglected organism-associated microorganisms in biodiversity and ecosystem function studies. Living aquatic organisms provide habitats for a wide variety of microorganisms, such as bacteria, fungi, algae, and protozoans. A rough estimate based on literature data indicates that bacteria densely colonize algae and zooplankton, reaching densities (i.e., number of bacteria per unit biovolume) far higher than in the ambient water. The relationship between these microbes and the base organisms can range from commensalism, parasitism to mutualism. With the exception of a few pathogens, surprisingly little is known about the ecology of microbial symbionts, such as their life cycle, their interactions with the hosts and adjacent microbes, and the evolution of such symbiotic relationships. However, recent whole genome sequence data suggest that symbiotic bacteria contribute substantially to the functional biodiversity in the aquatic world, influence the fitness of the host organisms, and thereby ecosystem functioning. Microhabitats within the higher organisms provide very different environmental conditions than the surrounding water, and they may therefore support the proliferation and activities of distinct microbial communities with important biogeochemical consequences. For example, earlier research suggested that the guts and feces of zooplankton and fish may support anaerobic microbial processes that otherwise cannot occur in oxygen-rich waters.

Recent advances in methodology such as profiling using microsensors allow researchers to characterize microhabitats within the higher organisms in unprecedented detail. Rapid development of single-cell and molecular techniques for phylogenetic and physiological analyses also offers enormous opportunities to study these symbionts at scales from a single gene to the whole community, and even their evolutionary history. New experimental approaches using genetically accessible model systems and individual-based modeling can also provide a mechanistic understanding of host-symbiont relationships.

This special issue brings together 11 articles that highlight new findings on biodiversity and functions of aquatic microbial symbionts, including microbial assemblages in close association with higher organisms.

The article by Wahl et al. (2012) presents a conceptual framework for studying the role of bacteria on the outer body of marine organisms, which represents a highly active interface between host and biofilm microbes. The authors show that biodiversity and functions of the attached microbiota are largely dependent on environmental parameters, and how the microbiota influences the host's ecology and health. The article by Bickel et al. (2012) focuses on ciliate epibionts of crustacean zooplankton in lakes. The authors show that ciliate epibiont abundance varies greatly between lakes and zooplankton species, respectively. Although the ciliate epibionts exhibited high grazing rates on free-living bacteria, their effects on the total bacterial abundance seemed to be rather low. Also, effects of epibionts on the physiology and development of their host require further study.

A cluster of three articles (Dziallas et al., 2012; Garcias-Bonet et al., 2012; McManus et al., 2012) addresses endosymbiotic relationships between microbes and higher organisms. The article by Dziallas et al. (2012) reviews knowledge on the diversity, ecological function, and evolution of ciliate symbionts and also points at several future research directions. The article by McManus et al. (2012) more specifically addresses chloroplast symbioses in marine ciliates. The authors discuss benefits and costs of chloroplast symbiosis for the ciliate *Strombidium rassoulzadegani*, and for the photo- and feeding-physiology of the ciliate. The paper by Garcias-Bonet et al. (2012) evaluates the biodiversity of endophytic bacterial communities on a marine angiosperm in the Mediterranean. The presence of bacterial endophytes greatly differed among locations and tissue types indicating a highly specialized bacterial endophyte community with potentially diverse and important functions for the angiosperm host.

Parasitic fungi in phytoplankton and parasitic dinoflagellates in marine copepods are the research foci in the papers by Sime-Ngando (2012) and Skovgaard et al. (2012), respectively. Both papers highlight the importance of parasites for plankton ecology and biodiversity, and planktonic food web dynamics. Several lines of evidence indicate that parasites have the potential to drive genetic diversity at the species, population, and community levels of pelagic ecosystems.

The next two articles evaluate the role of symbionts of an ascidian (Kühl et al., 2012) and a coral (Wangpraseurt et al., 2012) in photosynthetic activities in relation to environmental parameters such as irradiation, periods of anoxia, and physiology of the host. Kühl et al. (2012) focus on a chlorophyll *b*-containing symbiotic cyanobacterium and its photosynthetic activity, whereas Wangpraseurt et al. (2012) examine optical microniches in corals and provide evidence for the importance of such microniches for photobiology and stress response of the corals, as well as for the phenotypic and genotypic plasticity of the coral symbionts.

Finally, the articles by Dinasquet et al. (2012) and Rivera et al. (2013) report on the contributions of organism-associated bacteria to spatial heterogeneity of bacterioplankton activity and community composition in the sea. Dinasquet et al. (2012) show data indicating specific associations between certain bacterioplankton groups and their jellyfish host, whereas Rivera et al. (2013) highlight the role of ballast water for the dispersal of potentially pathogenic vibrio species.

In summary, these articles cover a range of symbiotic relationships in aquatic environments, pinpoint that these relationships are widespread, and that they conceivably play important roles for the health, adaptation, and evolution of the host organisms, and, thereby, for food web structure and ecosystem functioning. We hope that this special issue will stimulate more discussions and research on the fascinating subject of microbial symbiosis, which seems to be the rule rather than the exception in aquatic ecosystems.
